# A New Methodology to Determine the Orifice for Root Canal Configurations in First Permanent Molar Root and Canal Morphologies Using Micro-Computed Tomography

**DOI:** 10.3390/jcm13010071

**Published:** 2023-12-22

**Authors:** Casper Hendrik Jonker, Guy Lambourn, Anna Catherina Oettlé, Federico Foschi, Charlotte Theye, Ericka Noelle L’Abbé

**Affiliations:** 1 Faculty of Health, Peninsula Dental School, University of Plymouth Ground, Plymouth PL6 8BT, UK; guy.lambourn@plymouth.ac.uk (G.L.); federico.foschi@plymouth.ac.uk (F.F.); 2 Truro Dental Education Facility, Knowledge Spa, Royal Cornwall Hospital, Truro TR1 3HD, UK; 3 Anatomy and Histology Department, School of Medicine, Sefako Makgatho Health Sciences University, Pretoria 0204, South Africa; profoettle@gmail.com; 4 Forensic Anthropology Research Centre, Department of Anatomy, Faculty of Health Sciences, University of Pretoria, Pretoria 0031, South Africa; charlotte.theye@gmail.com (C.T.); ericka.labbe@up.ac.za (E.N.L.)

**Keywords:** micro-CT, cemento–enamel junction, Radix Entomolaris, C-shaped canal, mesotaurodont, fused roots, pulp floor, orifice, landmarks, root morphologies

## Abstract

Background: The distinction between where the pulp chamber ends and the root canal system begins is poorly defined within the existing literature. Methods: This paper aimed to describe a range of accurate methods to define the transition from pulp chamber to root canal in different first molar root morphologies using micro-focus computed tomography (micro-CT). Methods: The sample consisted of 86 mandibular and 101 maxillary first molars from the skeletal collections housed in the Department of Anatomy and Histology of the Sefako Makgatho Health Sciences University and the Pretoria Bone Collection. A stepwise approach using the cemento–enamel junction (CEJ) and dedicated landmarks was followed to create an automated cross-sectional slice. Results: Transition from pulp chamber to root canal could be accurately determined on maxillary and mandibular teeth. The occurrence of two separate roots in mandibular molars was 97.7%, with the remaining 2.3% having an additional disto-lingual root, with no mandibular molars displaying fused roots. In the maxillary molars, 92.1% had three separate roots and 7.9% displayed root fusion. Within this group, one tooth displayed a C-shaped root canal configuration and one a mesotaurodont-type morphology. Conclusion: The suggested methodology to determine orifice location was found to be appropriate in all morphological types.

## 1. Introduction

A sound knowledge of the root canal system is pivotal for diagnosis, treatment planning and successful execution of endodontic treatments [1,2,3]. There is also a close relationship between the external and internal morphologies of teeth. In most cases, the mandibular first molars have one mesial (M) and one distal (D) root, but additional roots can be present [4,5]. In maxillary first molars, the root morphology normally follows a three-rooted pattern with a mesio-buccal (MB), disto-buccal (DB) and palatal (P) root. These roots can be separate or fused, with prevalence varying between populations [2,6,7,8,9,10,11]. Reports indicate that, in cases where roots are fused, the internal root canal morphology can often be very complex [9,10,12].

The complexity of the pulpal morphology found in first molar teeth has led to a variety of classification systems being suggested by numerous authors over time [13,14,15,16,17,18]. It has been noted that the use of exact reference points in calculations by different authors is often unclear, confusing and subjective [18,19,20]. A common reference point such as the cemento–enamel junction (CEJ) cannot be used on a consistent basis as a clear separation point between pulp chamber and root canals to determine the location of their orifices for double and multi-rooted teeth. In single rooted teeth, comparisons are relatively standardised. The CEJ creates a natural separation between the pulp chamber and root canals and is considered a consistent and stable point of reference by many [18,21,22,23,24]. In multi-rooted teeth, however, the pulp floor usually extends further apically than the CEJ and corresponds to the root trunk [25,26].

The question, therefore, is which exact reference points will delineate the pulp floor necessary for the calculation of configurations of double and multi-rooted teeth in a consistent and repeatable manner. Recently, the Ahmed et al. classification was introduced and has been widely accepted for its application both clinically and in education [27]. This classification system has the ability to describe complexities and include fine details, using micro-focus X-Ray computed tomography (micro-CT) [22,27]. Micro-CT has been identified as the most suitable method to study root canal morphology because of its high resolution, which thus gives the ability to visualise complexities in the finest detail [28,29]. This scanning modality not only allows a three-dimensional (3D) observation of a tooth, but also emerged as a valuable tool to calculate root canal configurations [18,29,30,31,32,33,34,35].

The aim of this research is to describe a practical and consistent method to define landmarks on the pulp floor and the exact location of root canal orifices for standardisation purposes in first permanent molars with different root configurations. This study will also report for the first time on the number of roots, the presence of any additional roots and the prevalence of root fusion in permanent first molars in South African individuals of African descent.

## 2. Materials and Methods

### 2.1. Sampling Method and Approval

Micro-CT scans of human skulls from individuals of African descent with known sex and age originating from the Human Osteological Research Collection (HORC) housed in the Anatomy and Histology Department of the Sefako Makgatho Health Sciences University and the Pretoria Bone Collection (PBC), housed in the Department of Anatomy, University of Pretoria, South Africa [36,37] were studied. Prior to the investigation, ethical approval was obtained from the Research Ethics Committee of the Faculty of Health Sciences, University of Pretoria (Protocol number: 298/2020). Permission for research was given by family members in the case of a donation or is protected by the National Health Act 61 of 2012 in the case of unclaimed bodies.

### 2.2. Sample Selection

To minimise selection bias, a convenience sampling method was used. A total of 101 maxillary and 86 mandibular first molars from 87 individuals (48 males and 39 females) were included. The samples displayed slightly more teeth from the right side (53 maxillary and 44 mandibular molars) than the left (48 maxillary and 42 mandibular molars). A larger number of males (50 maxillary and 48 mandibular) than females (51 maxillary and 38 mandibular) were identified. The ages ranges were between 20 and 89 years.

### 2.3. Inclusion and Exclusion Criteria

Only teeth with intact roots were considered. Teeth with non-fully developed apices, incomplete roots, root fractures, coronal or radicular resorption, previous root canal treatments, restored with metal restorations or teeth where the pulp could not be adequately isolated after segmentation were not considered. Only scans which were deemed of adequate quality (highest resolution and without blurring) were included to allow proper isolation of the pulp.

### 2.4. Scanning Procedure

The skulls were scanned with a micro-CT scanner—the Nikon XTH 225L industrial CT system (Nikon Metrology, Leuven, Belgium) housed at the Micro-Focus X-ray Radiography and Tomography facility (MIXRAD) of the South African Nuclear Energy Corporation (Necsa, Pelindaba, South Africa). The spot size of the x-ray unit ranges between 0.001 and 0.003 mm (1–3 μm) and the translation table of the unit has a rotation accuracy to 1/1000th of a degree and a pixel size of 200 μ × 200 μ. The following parameters were used: 100 kV voltage, 100 mA current and 2.00 s exposure time per projection [38]. The Nikon CT Pro 3D version 4.4.3 software (Nikon Metrology) was used to reconstruct the final volumes with resolutions ranging between 40 and 74 µm.

### 2.5. Scan Alignment

To allow proper alignment of images and avoid oblique sections, and thus minimise possible bias, all the micro-CTs were re-oriented according to the CEJ of each tooth. The CEJ was chosen as it is present on all teeth as a continuous line between the crown and root of the tooth and therefore is commonly used as a standard plane for investigations in dentistry [39]. Using Avizo 2019 (Visualization Sciences Group Inc., Bordeaux, France) [40], a 3D imaging software, a set of landmarks was collected on the volume and placed on the CEJ of each tooth. A best-fit plane connecting all the landmarks was then automatically computed. This plane was then used as a reference to re-align the micro-CT image stacks.

### 2.6. Segmentation and Landmark Identification

To allow three-dimensional observation of each component of a tooth, namely the crown, enamel, root, pulp cavity and canals, a region-based semi-automatic segmentation procedure known as the watershed [41,42] was carried out in Avizo. Different colours were allocated to the enamel, dentine and pulp to allow proper differentiation. Segmentation can be described as the extraction of 3D regions of interest within the images by defining the contour of each structure. The region of interest of this study was the pulpal complex, which includes the pulp chamber, and root canals were segmented for each tooth [40], allowing for magnification and inspection of the morphology from all angles. A single operator with endodontic and 3D imaging/micro-CT experience (including the use of Avizo) was responsible for the segmentation of scans and placement of landmarks. Four landmarks (A, B, C, D) were placed for the mandibular teeth. Landmarks A and B were placed on the buccal surface, at the highest occlusal point of each root along the CEJ. Similarly, two landmarks (C and D) were placed on the lingual surface. In maxillary teeth, three landmarks were sufficient in most teeth: two (A and B) on the buccal surface (also at the highest occlusal point on the CEJ of the buccal roots) and one (C) on the palatal surface of the palatal root. In some maxillary teeth, where one point on the CEJ on the palatal surface extended further apically, a landmark was placed on either side of this extension (total of four). In the Avizo software, a cross-sectional plane connecting the landmarks was automatically created. By using this plane, landmarks D, E or F could subsequently be identified on the bifurcation/trifurcation zenith on the pulp floor. To confirm accuracy, a second operator, a specialist and Consultant in Prosthodontics with endodontic experience confirmed the exact locations of the landmarks. Where operators disagreed, a consensus was reached after discussion.

An in-depth description of orifice location with illustrative micro-CT images identified in different root and canal morphologies will follow in Section 3.1, Section 3.2, Section 3.3, Section 3.4, Section 3.5, Section 3.6 and Section 3.7.

## 3. Results

### 3.1. Mandibular First Molars: Two Rooted

**Figure 1:**
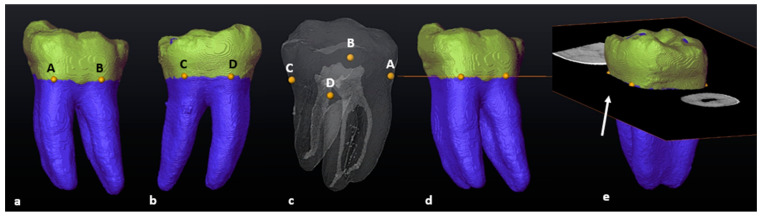
A 3D model of a two-rooted left mandibular first molar: (**a**) in buccal view (landmarks A and B: yellow dots); (**b**) in lingual view (landmarks C and D: yellow dots); (**c**) in semi-transparency and mesial view (landmarks–pulpal interface); (**d**) in buccal view with the cross-sectional slice (in orange); (**e**) rotated view of the slice (white arrow).

The following methodology could be repeated in 97.7% of the mandibular teeth (*n* = 84/86). Once the tooth was isolated, the transparency was manually reduced to hide the pulpal space. As defined in Section 2.6, landmarks A, B, C and D were placed on the buccal and lingual surfaces of each root (Figure 1a–c). By selecting the “Slice” and “Points To Fit” functions in Avizo, a cross-sectional slice was automatically positioned at the level of the four landmarks (Figure 1d,e).

**Figure 2:**
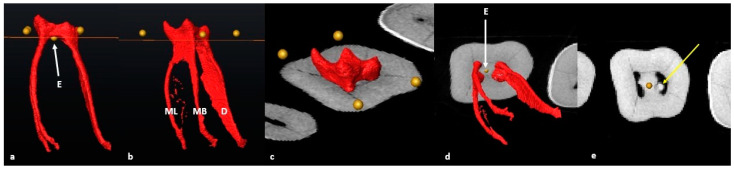
A 3D model of the extracted pulp: (**a**) buccal view of landmark E (yellow dot) and cross-sectional slice at the most superior point on the pulp floor; (**b**) mesio-buccal view of the pulpal complex including individual root canals (ML: mesio-lingual, MB: mesio-buccal and D: distal) and cross-sectional slice at the most superior point on the pulp floor; (**c**) occlusal view of the pulp chamber separated by the slice at landmark E; (**d**) apical view of the radicular pulp isolated by the slice at landmark E (white arrow); (**e**) cross-sectional view of the axial slice indicating the orifices of the mesial and distal canals. Note the pulp stone in the distal canal orifice (yellow arrow).

The pulpal space was then extracted, rotated and observed from different angles. The cross-sectional slice was manually moved apically by scrolling in an apical direction to the point where the slice crosses the pulp floor for the first time (landmark E) (Figure 2a–c). This location is the point of maximum convexity (bifurcation zenith) of the root canal bifurcation. The section of the root canal network coronal to the slice will be the pulp chamber and apical to the slice, the radicular pulp (Figure 2a–e).

**Figure 3:**
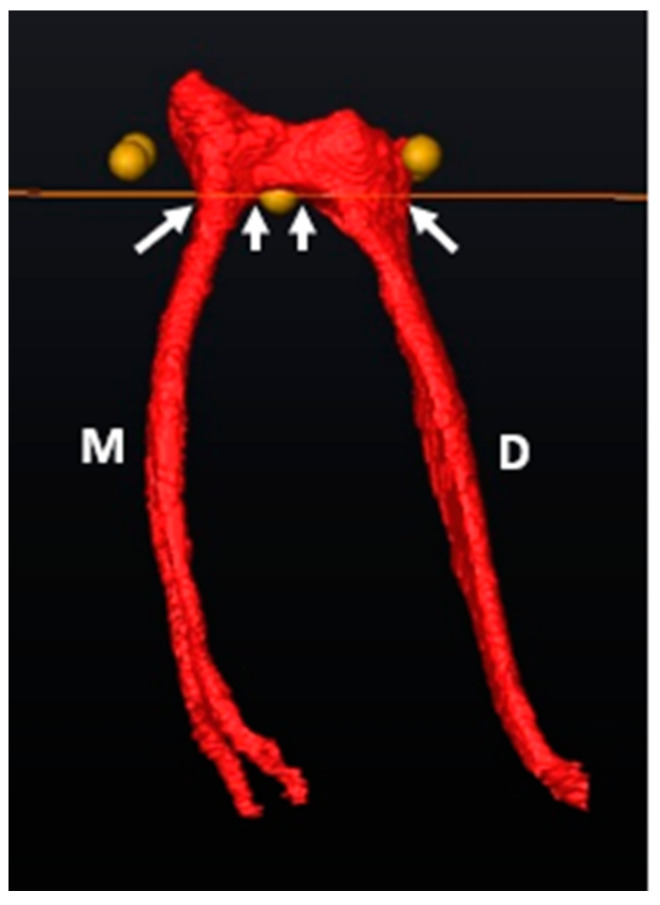
Orifice determination of the M and D canals using the cross-sectional slice and landmark E (bifurcation zenith). The white arrows below the cross-sectional slice indicate the outer and inner limits of the root canal orifice.

The orifice of the mesial and distal canal(s) is determined by observing the exact point where the slice crossed each canal from landmark E outwards from the bifurcation to the outer surface of each canal (Figure 3).

### 3.2. Mandibular First Molars: Three Rooted

**Figure 4:**
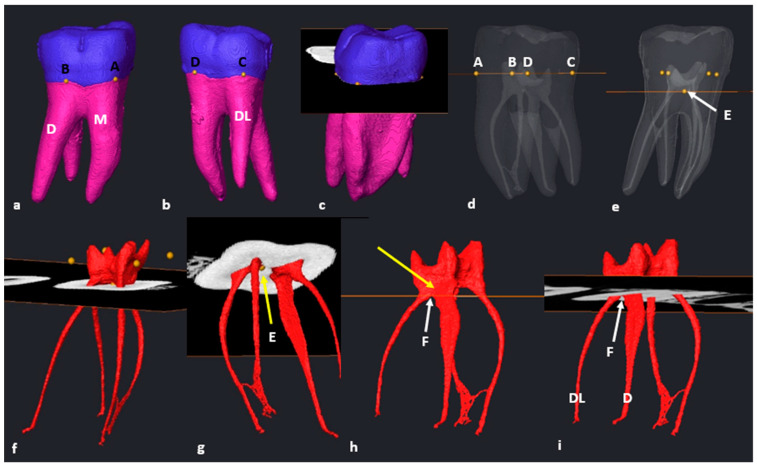
A 3D model of a three-rooted right mandibular first molar: (**a**) buccal view with landmarks A and B on the MB and DB root surfaces respectively; (**b**) lingual view with landmarks C and D on the DL and mesial roots, respectively; (**c**) cross-sectional slice viewed from mesial; (**d**) semi-transparency and lingual view (landmarks–pulpal interface). Note the location of the pulp floor in relation to the CEJ [18]; (**e**) cross-sectional slice at landmark E (white arrow); (**f**) mesio-buccal view of the separated pulp chamber and radicular pulp; (**g**) apical view from mesial illustrating the orifice(s) of the mesial root canals with slice positioned at landmark E (yellow arrow); (**h**) cross-sectional slice at the second bifurcation between the distal roots (landmark F, white arrow). The common chamber area is indicated by the yellow arrow; (**i**) apical view of the distal root canals and their orifices at landmark F (white arrow).

In the mandibular sample, two teeth with additional disto-lingual (DL) roots (*n* = 2/86: 2.3%) were present. The methodology for a three-rooted mandibular molar followed a similar pattern as described above, apart from a slight modification. Initially, four landmarks were placed in similar positions to two-rooted molars (Figure 4a–d), the cross-sectional slice was created and positioned at landmark E and the main root canal bifurcation point of all roots (Figure 4e–g). Once the slice is positioned at landmark E, the configuration of the mesial root can be calculated and indicates the orifice and starting point for configurations for the mesial canal(s). The slice was then manually moved to the point of maximum convexity between the distal and disto-lingual root canals by scrolling apically to locate landmark F (second bifurcation) (Figure 4h: white arrow). The slice located at the second bifurcation indicates the orifices and starting point for configurations for the distal canals, including the additional root (Figure 4h,i). The position of the slice at the second bifurcation also indicates the most apical point of the pulp floor. The chamber area between landmarks E and F is shared between the two distal root canals (Figure 4h: yellow arrow).

### 3.3. Maxillary First Molars: Three Separate Roots

**Figure 5:**
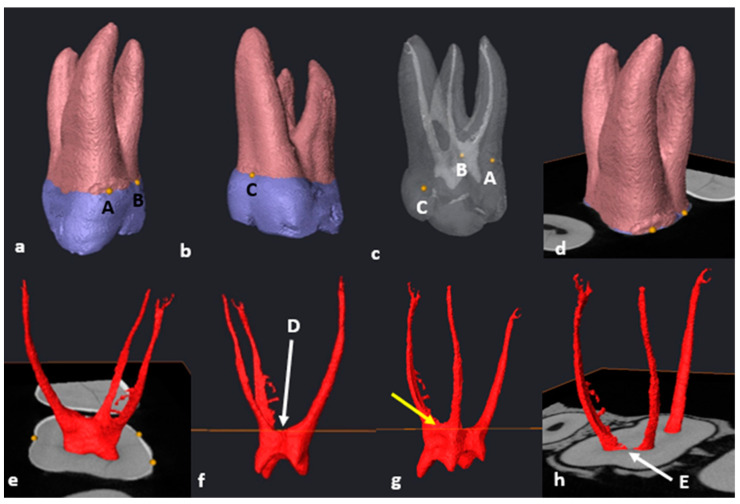
A 3D model of a maxillary first molar with three separate roots: (**a**) mesio-buccal surface view with landmarks A and B on the MB and DB roots, respectively; (**b**) mesio-palatal view of landmark C on the P root; (**c**) mesio-palatal view in semi-transparency and landmarks–pulpal interface; (**d**) cross-sectional slice viewed from mesio-buccal; (**e**) isolated pulp with cross-sectional slice at the level of landmarks A–C.; (**f**) distal view of the pulp with cross-sectional slice positioned at the most superior point of the pulpal trifurcation (landmark D, white arrow); (**g**) the common pulp chamber area between the buccal canals (yellow arrow); (**h**) cross-sectional slice positioned at the buccal bifurcation (most apical point of the pulp floor) and landmark E (white arrow).

Most maxillary teeth (*n* = 93/101; 92,1%) had separated MB, DB and P roots with root and root canal trifurcations in the coronal third. The transparency of the isolated tooth was reduced, and three landmarks (A–C) were placed at the most superior coronal location on the CEJ of each root. Landmark A was placed on the mesio-buccal root surface, landmark B on the disto-buccal root surface and landmark C on the palatal root surface (Figure 5a–d). The cross-sectional slice was created and positioned automatically at the level of the landmarks. Then, the slice was manually positioned to an apical position at the point of maximum convexity, where the slice crossed the pulp floor for the first time and the location of the pulpal trifurcation, defining the position of landmark D (Figure 5f). The root canal orifice and origin of configurations for the palatal canal can now be calculated by following the slice from landmark D outwards towards the palatal surface of the palatal canal (Figure 5f). A common part of the pulp chamber between the buccal canals with the slice at this position was noted (Figure 5g: yellow arrow). To determine the orifices for the mesial and distal canals, the cross-sectional slice was manually moved in an apical direction by scrolling down to the first point of contact with the buccal canal bifurcation, i.e., the most apical point of the pulp floor (landmark E) (Figure 5h).

### 3.4. Maxillary Molars: Variants in Mesial or Distal Bifurcations

**Figure 6:**
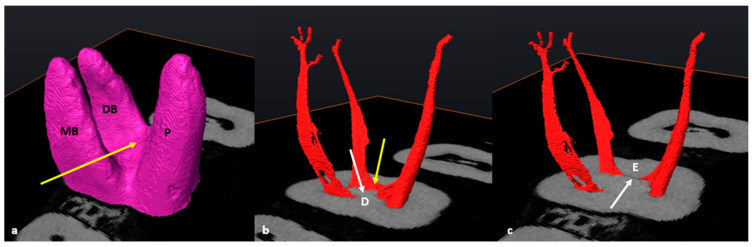
(**a**) Maxillary first molar with three separate roots (MB, DB and P) and an apical disto–palatal root bifurcation (yellow arrow); (**b**) cross-sectional slice at the most superior point of the pulp floor (landmark D, white arrow). The common chamber between the distal and palatal canals is indicated by the yellow arrow; (**c**) cross-sectional slice at the disto–palatal bifurcation (landmark E, white arrow).

Variants in mesial or distal bifurcations were noted in 2.2% of maxillary molars with three separate roots (*n* = 2/93). In some cases, it was noted that there were variants in the buccal, mesial and distal root and canal bifurcations. For instance, in one tooth, the disto–palatal root canal bifurcation was located more apically (*n* = 1/93; 1.1%) (Figure 6a). In cases like these, the suggested methodology will be similar with the following minor modification. The orifice(s) and starting point of root canal configurations in the mesial root can be determined with the cross-sectional slice positioned at landmark D (Figure 6b: white arrow). In this illustrated case, a common area of the pulp chamber is shared between the distal and palatal canals (Figure 6b: yellow arrow). The orifices of the distal and palatal canals could be determined by manually positioning the slice to landmark E, which is the first point of contact between the slice and disto–palatal root canal bifurcation (Figure 6c: white arow).

Another single tooth presented with a more apically positioned mesio-palatal root canal bifurcation (*n* = 1/93; 1.1%) The suggested methodology for orifice determination was similar, except that the distal canal orifice was determined with the cross-sectional slice positioned at landmark D. Furthermore, the orifices for the mesial and palatal canals were determined with the slice located on landmark E (first point of contact between the slice and the mesial root canal bifurcation).

### 3.5. Fused Roots

**Figure 7:**
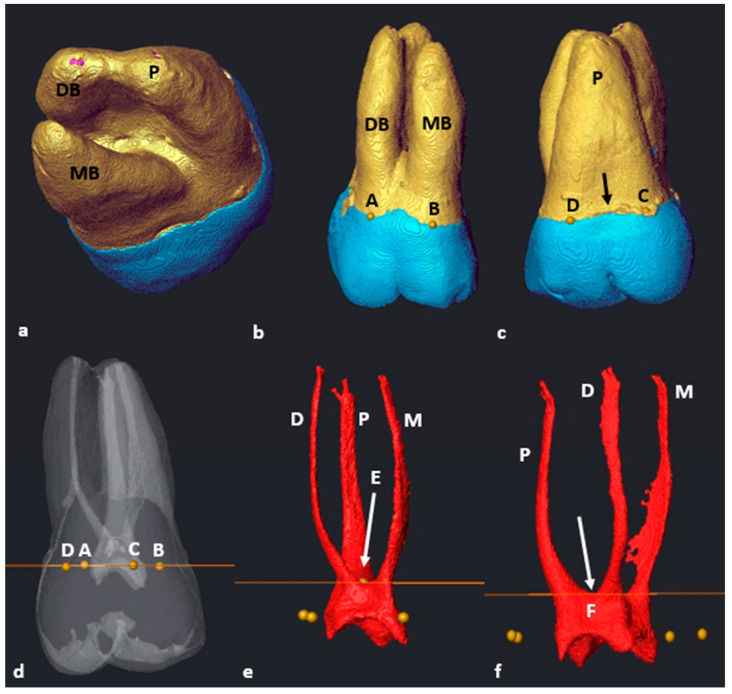
A 3D model of a fusion type 3 two-rooted maxillary first molar: (**a**) apical view with fused DB and P roots [12]; (**b**) buccal view with landmarks A and B on the DB and MB roots, respectively; (**c**) palatal view with landmarks C and D on the P root. Note the apex created by the CEJ (black arrow); (**d**) tooth in semi-transparency with landmarks–pulpal interface and cross-sectional slice (in orange); (**e**) buccal view of the extracted pulp and cross-sectional slice (in orange) at landmark E (pulp chamber and radicular pulp separation, white arrow); (**f**) disto-buccal view of the extracted pulp with landmark F (white arrow).

Maxillary molars with fused roots according to the descriptions by Zhang et al. (2014) were found in a small number of teeth (*n* = 8/101; 7.9%) [12]. The methodology for seven of these teeth (87.5%) followed similar steps as the other maxillary first molars with three separate roots, as previously described (Figure 7a–f). No root fusion was noted in mandibular first molars.

In a single tooth (*n* = 1/8; 12.5%) which displayed root fusion, one area of the CEJ was located more apically on the palatal surface (Figure 7c, black arrow). In such cases, a landmark can be placed either side of the more apically extending portion of the CEJ (C and D) (Figure 7c). This is also applicable for the maxillary first molars with three separate roots, where this morphology occurred in 15.1% (*n* = 14/93) of the sampled separate-rooted teeth. The positioning of the slice, identification of trifurcations and bifurcations and determination of orifices were the same as described above for molars with three separate roots.

### 3.6. C-Shaped Canals

**Figure 8:**
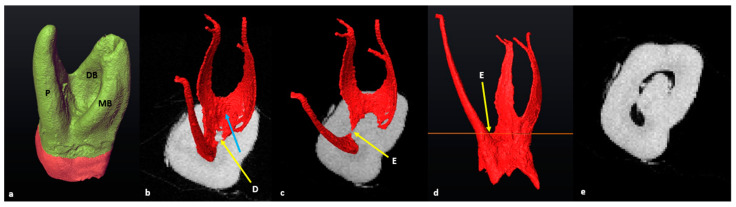
A 3D model of the maxillary first molar with a C-shape canal configuration (**a**) mesio-apical view with fused MB, DB and P roots; (**b**) cross-sectional slice at landmark D (yellow arrow). The blue arrow indicates the ribbon-shaped canal in the coronal third and common orifice of the MB and DB canals; (**c**) cross-sectional slice at the distal bifurcation point (landmark E, yellow arrow); (**d**) mesial view of the extracted pulpal complex with cross-sectional slice at the level of landmark E; (**e**) cross-sectional slice at landmark E, illustrating a C2-type canal shape in the coronal third [43].

The C-shaped type of pulpal configuration was observed in a single maxillary first molar (*n* = 1/101: 0.99%) where the MB and DB roots were fused. The P root remained separate according to the fusion ratio determined by Zhang et al. (2014) [12]. No C-shaped canals were identified in the mandibular molars. The cross-sectional slice at landmark D (maximum convexity) indicates the orifices’ locations and point of origin for configurations for the mesial and distal root canals (Figure 8b, yellow arrow). In Figure 8, the mesial and distal canals displayed a large continuous common canal in the coronal third (Figure 8b, blue arrow) shared between the mesio-buccal and disto-buccal canals. By scrolling down in an apical direction, the cross-sectional slice was then positioned at landmark E (the most apical location of the pulp chamber floor) and the most superior point on the disto–palatal bifurcation area (Figure 8c,d). The location of the palatal canal orifice could now be determined. At landmark E, the canals displayed a C-shape configuration similar to a C2-type configuration [43,44]. There was a ribbon-shaped mesial, buccal and distal canal space and a separate palatal canal (Figure 8e) [43].

### 3.7. Taurodontism

**Figure 9:**
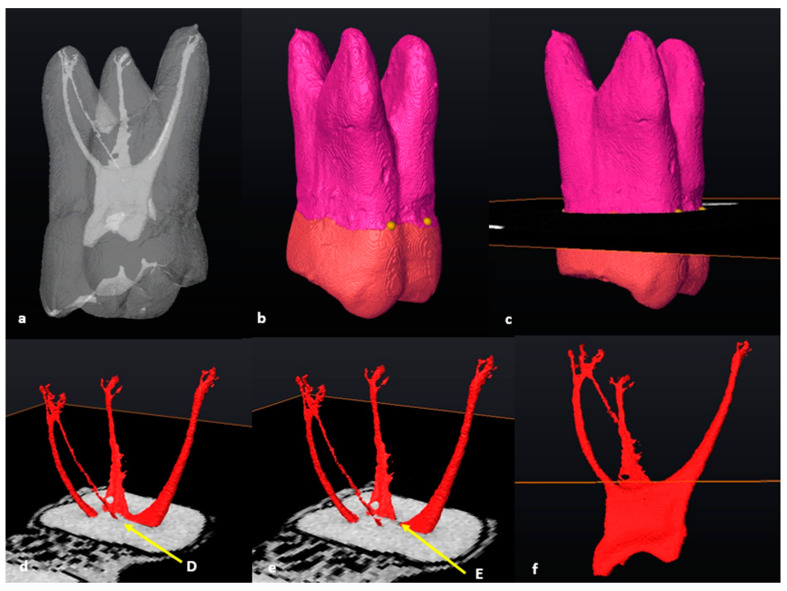
A 3D model of a maxillary first molar with mesotaurodontic traits: (**a**) in semi-transparency; (**b**) disto-buccal view with buccal landmarks; (**c**) cross-sectional slice positioned at the level of the landmarks; (**d**) apical view of the cross-sectional slice at landmark D (yellow arrow) with the extracted pulp; (**e**) apical view of the cross-sectional slice positioned at landmark E (yellow arrow); (**f**) pulp chamber and radicular pulp separated at the most apical point of the pulp floor by the cross-sectional slice (in orange).

An in-depth calculation of taurodontism (bull-like tooth) did not form part of the initial investigation. However, a maxillary molar with mesotaurodontic traits as described by Hasan [45] was identified (*n* = 1/101; 0.99%). The methodology to determine the pulpal configuration followed a similar pattern, as described in the section on maxillary molars, where the mesial or distal bifurcations were on different levels compared to the buccal one (Figure 9). In this particular molar, the disto–palatal root and canal bifurcations were also positioned further apically.

### 3.8. Number of Roots

In mandibular molars, most teeth had two separate roots: one M and one D (*n* = 84/86, 97.7%). Only two teeth had an additional DL root and were classified as Radix Entomolaris (RE) (*n* = 2/86, 2.3%). In one of these two teeth, the additional root had no curvature and was classified as type I according to the Ribeiro and Consolaro classification [46]. The other tooth displayed an additional root with a coronal curvature with a straight continuation to the apex and was classified as type II [46].The mandibular sample did not contain teeth with single roots, fused roots or more than three roots.

In maxillary molars, most teeth had three separate roots: a MB, a DB and P (*n* = 93/101, 92%). The eight remaining teeth had three roots, but the MB, DB or P roots were fused (*n* = 8/101; 7.9%). According to the criteria by Zhang et al. [12], most fusions were type 3 with fused DB and P roots (*n* = 5/8, 62.5%); followed by type 1 with fused MB and DB roots (*n* = 2/8; 25%); and finally type 2, with fused MB and P roots (*n* = 1/8; 12.5%). No other types of fusion were noted.

## 4. Discussion

In a recent critical analysis of laboratory and clinical research methodology, researchers were encouraged to find a universal consensus in their approaches and terminology followed when calculating root canal configurations [20]. The authors stated that it was important to find consensus to allow comparison between studies. Therefore, the decision was made to describe a stepwise approach to determine important landmarks for configuration purposes.

Ahmed et al. (2022) suggested clear guidelines for some terminological aspects related to root and canal anatomy. One of these included the use of the point of termination on the pulp chamber floor, and the emerging point or orifice of the root canal. The article also stated that the transition from the pulp chamber to the individual root canals seems to be an area that is particularly problematic due to lack of standardisation. Currently, the description of a root canal orifice according to the American Association of Endodontists is the opening which leads from the pulp chamber into a root canal, especially in a tooth with multiple canals [21]. Ahmed et al. (2017) described the orifice as the opening of the canal system at the base of the chamber where the canal begins and is normally located at, or just apical to, the cervical line [22]. Therefore, the question that needs to be addressed is where exactly the location of the orifice is, in relation to the pulp chamber and cervical line.

In recent articles, Ahmed and colleagues (2018, 2021) posted visual presentations of certain landmarks on the pulp floor and orifices (2018; Figure 4; 2021: Figures 13 and 14). These images illustrated molars from longitudinal and cross-sectional viewpoints but exact descriptors to locate these landmarks were not provided [18,47]. One image (Figure 13 in the 2021 article) illustrated a cross-sectional slice similar to the one in this study, but the methodology to consistently create this slice and the landmarks was not provided. Although the current authors agree with this observation, it must be noted that the lack of clear, stable landmarks and a standardised methodology to determine the apical extend of the pulp chamber with a cross-sectional slice could potentially cause discrepancies between root canal configuration reports. A cross-sectional slice positioned without reliable and repeatable landmarks might create horizontal planes based on subjective views or uneven planes, and thus potentially incorporate inaccuracies into calculations. To the best of the authors’ knowledge, this study is the first practical and consistent description aimed at reducing discrepancies. The methodology suggested allows the identification of reliable landmarks on the pulp floor and thus determines the accurate location of root canal orifices in maxillary and mandibular permanent first molars. The methodology presented in this study proposes to build on the work by Ahmed et al. (2017, 2020, 2022) by adding identifiable landmarks and describing a stepwise approach allowing consistency [20,22,27].

The approach described could be performed on all the teeth of the sample. In most mandibular molars (*n* = 84/86; 97.7%), landmark E and the automatically positioned cross-sectional slice could be used to determine the orifices of the distal and mesial root canals by following the slice either mesially or distally outwards from point E. For the two remaining mandibular first molars (*n* = 2/86; 2.3%), an adaptation of the methodology was suggested. Indeed, these two molars had additional roots located on the disto-lingual surface, which is known as Radix Entomolaris (RE). These additional roots are quite rare and were first described by Bolk (1915) [48]. The root and root canal morphology of these three-rooted mandibular molars required an additional step (similar to maxillary molars with three separate roots). The samples from this study included no single or four-rooted teeth, which can be considered as a limitation. However, it can be speculated that other three-rooted or even four-rooted variants can be approached in a similar way.

### 4.1. Mandibular Molars

Almost ninety-eight per cent (97.7%) of the mandibular molars in this study sample had a distinct M and D root. Only two teeth had three roots (2.3%). This finding is in line with results from previous work, which stated that the maximum incidence of an additional root in African populations is 3% [49]. Tredoux et al. [11] recently also investigated a South African sample and found that 98% of teeth had two roots and only 1% had three roots. The figure is lower than in this investigation, however, in the Tredoux et al. study, the population group was not specified. In an Ugandan study, where extracted teeth from individuals of African descent were investigated, Rwenyonyi et al. [50] did not find any three-rooted first molars, and all teeth had two distinct M and D roots.

Globally, incidences vary according to population affinity, but similar findings to this investigation were noted in a Turkish population (95.8% were two rooted and 2.06% three rooted) [51]. The aetiology behind this type of morphology has been a focus of discussion for years. It has been speculated that external factors during odontogenesis and population-specific genetic factors could play a role [46,52]. The presence of this additional root has clinical significance and careful observation of diagnostic tools, whether radiographs or three-dimensional (such as with cone-beam computed tomography (CBCT)), is important to avoid the possibility of untreated root canals and ultimately treatment failure [52].

### 4.2. Maxillary Molars

The majority of the maxillary first molars in this study displayed root morphology of either three separate (92.1%) or fused (7.9%) roots. This finding is similar to a CBCT study recently performed on a South African sample from the Gauteng province in which 91% of maxillary first molars had three separate roots and 8% had two fused roots [53]. The study, however, did not specify the population affinity of the individuals, but it can be speculated that most of them were Black South Africans. Many authors highlighted that teeth with fused roots can harbour a complex internal root canal morphology, which can be found across the length of the root [9,10,12]. This complexity will have clinical implications in the fact that endodontic treatment planning and execution could be challenging.

Complex root canal morphology has been mentioned as an important factor that could influence treatment outcomes [2]. In the current research, most root fusions (62.5%; *n* = 5/8) displayed a type 3 configuration according to Zhang et al. [12], i.e., fusions between the DB and P roots. This result agrees with another South African study, in which the authors observed that most of the fusions were of type 3 [53]. An Arabian study [54] also using the Zhang classification criteria, found that 6.8% of maxillary first molars displayed root fusion between the DB and P roots (4.5%). In a Portuguese sample, 7.1% of roots were fused, of which 6% were Zhang type 3 [9]. In contrast, in a study from Uganda, more fusions occurred between the MB and P roots and no DB and P roots were fused [6].

Within the maxillary sample of this study, one molar was identified with typical traits of taurodontism (*n* = 1/101; 0.99%). In this type of tooth, the pulp morphology is altered as the pulp chamber and floor are situated more apically, which can create challenges during endodontic treatments [55]. The reported prevalence ranges between 0.57% and 4.37% [56,57], which agrees with the results of this study (0.99%). The methodology followed to identify landmarks and orifices was identical to the other morphologies, except that the pulp chamber floor and root and canal bifurcations were positioned more apically.

To locate the orifices of maxillary first molar root canals, slight modifications of the protocol were required for some teeth (fused roots, C-shaped canals and variant levels of bifurcation). In most molars (*n* = 98/101, 97%) including both separate or fused roots, the buccal root canal bifurcations (MB-DB) were more apical compared to the mesial and distal bifurcations (MB-P and DB-P). In a small number of teeth, the distal or mesial root and canal bifurcations were more apically positioned than the buccal bifurcations (*n* = 3/101, 3%) (see Figure 6). In these types of root and canal morphologies, an apical positioning of the cross-sectional slice to the distal or mesial root canal bifurcation was required to determine the orifice location of the respective mesial, distal and palatal canals.

As the sample of maxillary teeth did not contain single- or four-rooted teeth, it can only be speculated that the described methodology will be similar, or adjustable. It must be emphasised that, although the type of fusion is not decisive, the presence of fusion in first molars has clinical significance and clinicians should be mindful of their presence. Despite tremendous advances, it is virtually impossible for current root canal instrumentation to reach all areas of the root canal system, in particular complexities. As stated previously, teeth with fused roots can harbour a complex internal morphology and a vigorous irrigation protocol should be followed to ensure maximum disinfection. Complex root canal systems, therefore, pose a therapeutic risk as chemical disinfection rather than mechanical could be detrimental for long-term survival of an affected tooth [57,58].

Although not a common finding, C-shaped canal configurations can be present in maxillary first molars [59]. In a Belgium population, researchers analysed clinical records (*n* = 2175) over a 10-year period and reported a prevalence of 0.09% [60]. In a study focusing on Chinese individuals living in Taiwan (*n* = 305) and using a clearing technique, 0.3% of teeth displayed this bizarre type of root canal configuration [61]. A single tooth displaying this type of morphology was identified in the current study which is approximately 1% (*n* = 1/101) of the sample size. This result is higher than in the Belgium and Taiwanese studies and it could be speculated that the use of a high-resolution scanning technique (micro-CT) or differences between sample sizes could have played a role. The root and root canal configurations of teeth displaying these types of root and pulpal configurations can be extremely complex [43,62]. The tooth also displayed a type 1 root fusion (MB and DB root fusion—see Figure 8a) [12] with a high coronal distal root and root canal bifurcation. The suggested methodology for this single tooth is similar to the other maxillary first molars, but for configuration calculations, a common orifice will be shared between the mesial and distal canals. In cases where researchers use the Ahmed et al. classification system [22], the configuration will be shared and indicated with a double slash (//). According to the classification modification suggested by Fan et al. (2007), this tooth displayed a C1 type configuration from the most superior point on the pulp (maximum convexity) to the distal root canal bifurcation. The pulp is continuous from mesial, extended over the buccal surface onto the distal and connected to the palatal canal. At the most apical point of the distal bifurcation, the pulp configuration changed into a type C2 to the apical third for the two fused buccal roots [43].

Interestingly, another study found a similar type of C-shaped root and canal morphology but on a maxillary second molar. The two buccal roots were also fused with a C-shaped buccal root canal system and a separate palatal canal [63]. The determination of landmarks on this challenging morphology requires careful investigation of the pulp floor and bifurcating areas. Considering the complexity of these teeth, the suggested methodology proposed in this paper might need modification for other teeth or other types of C-shaped configurations. To the best of the authors’ knowledge, there are no other micro-CT studies reporting on C-shaped configurations in maxillary permanent first molars in South Africans of African descent.

The limitations of this study are that only first molars were included, meaning that the proposed methodology could not be tested against other teeth, but it performed well on the teeth included. Also, a larger pool of assessors may have added benefit to confirm landmark locations. A relatively small sample size could also be considered a limitation as a larger sample may have identified more varied root forms. However, similar sample sizes were used by other investigators [5,35,62]. As the study only included South Africans from the northern Gauteng province, it is not known to what extent the methodology will be applicable to other groups within or beyond South Africa. Finally, the samples from this study included no single- or four-rooted teeth, which can be considered as a limitation as the morphology of other teeth may vary. However, it can be speculated that the methodology used could be applied to other multi-rooted teeth in a similar fashion.

## 5. Conclusions

This article provides an objective and consistent methodology to define the exact location of root canal orifices in all roots of maxillary and mandibular permanent first molars with different root morphologies. The use of a standard approach to classify root canal configurations will assist the clinician with the challenge of diagnosis and treatment planning for more complicated cases. The operator will have an accurate perception of the root canal complex from orifice to apical exit in each root. This approach provides a precise starting location for configuration, particularly when the Ahmed et al. classification system is used. The proposed methodology will allow more standardised results between studies and is highly adaptable to the type of tooth. By using the CEJ, a stable standard point for placing set landmarks [39], to create automatically a cross-sectional slice as reference, a more accurate and objective description of the root canal orifice in maxillary and mandibular permanent first molars might be: “the entrance of a root canal at the most apical point on the pulp chamber determined by a cross-sectional plane positioned at set landmarks on the pulp floor”. The precise location of the cross-sectional plane at allocated positions is critical to determine exact locations on the pulp floor, including root canal bifurcations or trifurcations. This plane should be considered as the central component to determine root canal orifices in future projects.

## Data Availability

The data presented in this study are available from the corresponding author upon request. The data are not publicly available due to ethical reasons.
